# Effectiveness and safety of belimumab and telitacicept in active systemic lupus erythematosus: a retrospective cohort study

**DOI:** 10.3389/fimmu.2026.1927886

**Published:** 2026-09-21

**Authors:** Yi Shao, Yidan Song, Yingying Gu, Yan Li, Yanfeng Chen, Rui Jing, Haixia Cao, Zhanyun Da

**Affiliations:** 1 Department of Rheumatology and Immunology, Affiliated Hospital of Nantong University, Nantong, China; 2 Department of Rheumatology and Immunology, Nantong Tongzhou Eighth People’s Hospital, Nantong, China; 3 Medical School of Nantong University, Nantong, China

**Keywords:** active systemic lupus erythematosus, belimumab, IPTW, LLDAS, telitacicept

## Abstract

**Background:**

Direct comparative evidence on the effectiveness of telitacicept versus belimumab in active systemic lupus erythematosus (SLE) remains limited. This study aimed to compare their effectiveness and safety in patients with active SLE.

**Methods:**

This retrospective cohort study included 91 patients with active SLE treated at Affiliated Hospital of Nantong University between July 2021 and June 2024. Eligible patients received conventional therapy and were treated with belimumab (n=45) or telitacicept (n=46) for 24 weeks. Inverse probability of treatment weighting (IPTW) was applied to balance baseline covariates. The primary outcome was the proportion of patients achieving LLDAS at week 24.

**Results:**

Telitacicept was associated with significantly better outcomes than belimumab in achieving LLDAS at week 24 (30.92% vs. 11.35%, *P* = 0.026) and SRI-4 responses at weeks 12 and 24 (both *P* < 0.01). The steroid-sparing effect also favored telitacicept, as evidenced by higher proportions of patients achieving prednisone reduction to ≤5 mg/d (40.99% vs. 18.72%, *P* < 0.05) and ≤7.5 mg/d (52.14% vs. 27.25%, *P* < 0.05). Additionally, telitacicept led to a significantly greater reduction in SLEDAI-2K score from baseline at both time points. In contrast, no significant between-group differences were observed in serological indicators. In exploratory multivariable analysis, telitacicept treatment (OR 4.975, *P* = 0.006), higher SLEDAI-2K score (OR 1.316, *P* = 0.016), and shorter disease duration (OR 0.914, *P* = 0.031) were independently associated with SRI-4 response at week 24. Both treatments showed numerically low adverse event rates, with no serious events reported.

**Conclusions:**

Telitacicept was associated with higher weighted response rates than belimumab in this single-center retrospective cohort of patients with active SLE after the reported adjustment.

## 1 Introduction

Systemic lupus erythematosus (SLE) is a chronic, multisystem autoimmune disease characterized by immune dysregulation, the production of diverse autoantibodies, and widespread inflammatory responses (1). Clinical manifestations are highly heterogeneous, with potential involvement of the skin, joints, kidneys, hematologic system, nervous system, and cardiovascular system; in severe cases, SLE can result in organ dysfunction or failure (2). SLE predominantly affects women of childbearing age, with a male-to-female ratio of approximately 1:9 (3). The global prevalence is about 43.7 per 100,000 people (3, 4), while in China, the prevalence is around 70 per 100,000 and shows an increasing trend (5). The natural course of SLE is characterized by alternating periods of remission and relapse. Patients with poorly controlled disease activity may develop irreversible organ damage, which significantly impacts their quality of life and long-term survival (6, 7). Although glucocorticoids, antimalarial agents, and conventional immunosuppressants remain the mainstay therapies for SLE, a considerable proportion of patients respond poorly to these treatments, or develop steroid dependence or steroid resistance, and may even face serious adverse effects from long-term high-dose glucocorticoid exposure, including infections, osteoporosis, and metabolic disturbances (8). Therefore, the development and optimization of safer and more effective targeted therapeutic strategies represent a critical unmet clinical need in the field of SLE.

In recent years, significant breakthroughs have been achieved in biologics targeting key immune pathways in SLE. Belimumab, as the first biologic approved for SLE treatment, is a fully humanized IgG1λ monoclonal antibody that specifically binds to and neutralizes soluble B lymphocyte stimulator (BLyS/BAFF), thereby inhibiting B-cell survival, proliferation, and differentiation into plasma cells, and consequently reducing the production of autoantibodies (9). Multiple clinical studies have demonstrated that adding belimumab to standard therapy significantly increases the SLE Responder Index (SRI)-4 response rate, reduces disease activity, decreases cumulative corticosteroid dosage, and delays severe relapse (9–11). However, approximately half of patients still fail to achieve a satisfactory clinical response with belimumab (12), or experience a decline in efficacy over time, underscoring the need for switching to other therapeutic regimens or transitioning between biologics.

Unlike belimumab, which targets only BLyS, telitacicept is a dual-target recombinant transmembrane activator and calcium modulator and cyclophilin ligand interactor (TACI)-Fc fusion protein with a broader mechanism of action (13). This protein consists of the extracellular ligand-binding domain of the human TACI receptor fused with the Fc fragment of human IgG1, enabling it to neutralize both BLyS and a proliferation-inducing ligand (APRIL), two key cytokines. By competitively binding to BLyS and APRIL, telitacicept blocks their interaction with B-cell surface receptors, thereby inhibiting B-cell overactivation and plasma cell differentiation, and reducing the production of pathogenic autoantibodies and immune complexes (14). Telitacicept received conditional marketing authorization for the treatment of SLE in China in 2021 (13). Multiple clinical trials have confirmed its favorable efficacy and safety profile in patients with active SLE (15–17). However, despite the clinical availability of both telitacicept and belimumab, head-to-head comparative studies of these two biologic agents in the treatment of active SLE remain limited. To address this limitation, we conducted a retrospective cohort study to compare the effectiveness and safety of telitacicept and belimumab in real-world settings among patients with active SLE who had persistently high disease activity despite receiving conventional therapy.

## 2 Methods

### 2.1 Study design and patients

This single-center, comparative, retrospective cohort study included 91 patients with active SLE between July 2021 and June 2024 at the Department of Rheumatology and Immunology, Affiliated Hospital of Nantong University. Patients were grouped according to the biological agent they received: belimumab (n=45) or telitacicept (n=46). The choice between telitacicept and belimumab was based on patient affordability, dosing convenience, treatment duration preferences, and the physician’s comprehensive clinical judgment. The study received approval from the ethics committee of Affiliated Hospital of Nantong University (ethical approval number: 2021-Y048-01). Written informed consent was obtained from all patients.

The inclusion criteria were as follows: patients who met 2012 Systemic Lupus International Collaborating Clinics (SLICC) or the 2019 European League Against Rheumatism (EULAR) and American College of Rheumatology (ACR) classification criteria for SLE; had received conventional treatment with glucocorticoids, antimalarials and immunosuppressants, and still had high disease activity—defined as positive anti-dsDNA antibody, low complement, and SLE Disease Activity Index 2000 (SLEDAI-2K) score ≥8—or active lupus nephritis (LN); had no prior treatment with biological agents; received standardized treatment with belimumab (10 mg/kg once every 2 weeks for the first 3 doses, then once every 4 weeks) or telitacicept (160 mg once weekly). Key exclusion criteria included the following: severe dysfunction of vital organs or malignant tumors; coexistence of other rheumatic diseases (e.g., rheumatoid arthritis, Sjögren’s syndrome, psoriasis, ankylosing spondylitis); pregnancy or breastfeeding; seriously incomplete clinical data; and active hepatitis B or active tuberculosis. Seriously incomplete clinical data was defined as complete absence of post-treatment follow-up assessments, including SLEDAI-2K and core laboratory examinations, which precluded outcome evaluation. Among patients excluded for this reason, 5 patients (belimumab, n=3; telitacicept, n=2) discontinued or switched therapy due to economic constraints and did not return for any subsequent follow-up visits after treatment initiation. All 91 included patients completed the 24-week follow-up period. No patient discontinued treatment, switched to the alternative biologic, or was lost to follow-up before the week-24 assessment. In addition, no patient required rescue therapy during the observation period.

### 2.2 Data collection and study outcomes

From medical records, we obtained patient demographic characteristics (e.g., age, sex, and disease duration), details of treatment exposures (including concomitant medications and dosages), and other clinically relevant information. Baseline and follow-up assessments comprised serological measures (complement levels, immunoglobulin concentrations, and anti-dsDNA titers), daily glucocorticoid dosages, and additional clinical findings. The SLEDAI, PGA and BILAG scores were assessed to quantify disease activity and were extracted from our prospectively maintained SLE cohort database for this retrospective analysis. Adverse events during the observation period were collected from routine clinical notes.

The index date was defined as the date of the first dose of belimumab or telitacicept. Baseline measurements were defined as the most recent clinical assessments performed within 30 days prior to the index date. Week-12 and week-24 outcomes were defined using the closest visit data within windows of 12 weeks ± 7 days and 24 weeks ± 7 days, respectively, after the index date.

The primary outcome was the proportion of patients with active SLE achieving LLDAS at week 24 after treatment with belimumab or telitacicept. Other secondary outcomes included the proportion of patients achieving SRI-4 response at weeks 12 and 24; the proportion of patients achieving prednisone dose reduction to ≤5 mg/d and ≤7.5 mg/d at week 24; and improvements in SLEDAI-2K and serological indicators. Exploratory outcomes included prespecified subgroup analyses stratified by major organ involvement (LN vs. non-LN) and by hematological involvement. Additional exploratory analyses were performed to examine factors associated with SRI-4 response.

LLDAS was defined as SLEDAI-2K ≤4 with no major organ activity, no new disease activity, PGA ≤1, prednisone dose ≤7.5 mg/day, and stable maintenance immunosuppressive therapy (18). SRI-4 response was defined as a reduction of ≥4 points in the Safety of Estrogens in Lupus Erythematosus National Assessment-SLEDAI (SELENA-SLEDAI) score, no new British Isles Lupus Assessment Group (BILAG) A or >1 new BILAG B organ domain scores, and no worsening in the PGA score (<0.3-point increase) (19). Complete renal response (CRR) and partial renal response (PRR) were utilized for renal assessment of patients with LN (20). CRR defined as the simultaneous achievement of: 24-h urine protein (24hUP) <0.5 g or urinary protein to creatinine ratio (UPCR) <0.5 g/g; and estimated glomerular filtration rate (eGFR) decrease ≤10%-15% from baseline or eGFR ≥90 mL·min^-1^·(1.73 m^2^)^-1^. PRR was defined as the simultaneous achievement of: ≥50% reduction in 24hUP from baseline to <3.5 g, or ≥50% reduction in UPCR from baseline to <3.0 g/g; and eGFR decrease ≤10%-15% from baseline or eGFR ≥90 mL·min^-1^·(1.73 m^2^)^-1^.

### 2.3 Statistical analysis

Statistical analyses were performed using SAS (version 9.4). Multivariable logistic regression was used to calculate propensity scores (PS), with the model incorporating age, sex, SLEDAI-2K, PGA and BILAG as covariates. To reduce the influence of potential confounders when comparing the two treatment groups, inverse probability of treatment weighting (IPTW) was applied. Specifically, the PSs were weighted by the ratio of patients treated with telitacicept to all patients/PS in the telitacicept group and the ratio of patients treated with belimumab to all patients/(1-PS) in the belimumab group. The balance of baseline characteristics between groups was evaluated before and after IPTW adjustment using standardized mean differences (SMD). Additional methodological details on the PS model are available in the **Supplementary Materials**.

Continuous variables were presented as mean ± standard deviation (SD) or median with interquartile range (IQR), while categorical variables were expressed as percentages. For comparisons of clinical characteristics between groups, the Chi-square test or Fisher’s exact test was used for categorical variables, and analysis of covariance (ANCOVA), the independent samples t-test, and the Mann-Whitney U test were used for continuous variables. No missing data were observed for the primary outcome (LLDAS) and SRI-4. For baseline characteristics and other secondary outcomes, complete-case analysis was applied. Missing data for each variable at each visit are summarized in **Supplementary Table 1**. Univariate and multivariate logistic regression models were used to analyze the factors associated with achieving SRI-4 at week 24. Variables that were significant in the univariate analysis were incorporated into the multivariate logistic regression model. Two-sided *P* < 0.05 were considered statistically significant.

## 3 Results

### 3.1 Baseline characteristics before and after propensity score-based IPTW

Between July 2021 and June 2024, a total of 1547 patients with SLE attended our center. After applying the inclusion and exclusion criteria, 91 eligible patients were included in this retrospective cohort study, with 45 patients receiving belimumab and 46 patients receiving telitacicept for over 24 weeks (**Figure 1**). The baseline demographic and clinical characteristics of the two treatment groups are summarized in **Table 1**. The mean age was 36.22 ± 11.49 years in the belimumab group and 34.48 ± 10.83 years in the telitacicept group. Female patients accounted for 91.11% (n=41) of the belimumab group and 97.83% (n=45) of the telitacicept group. The median disease duration was 9.00 years (IQR, 4-15) and 10.50 years (IQR, 4.5-16), respectively.

**Figure 1:**
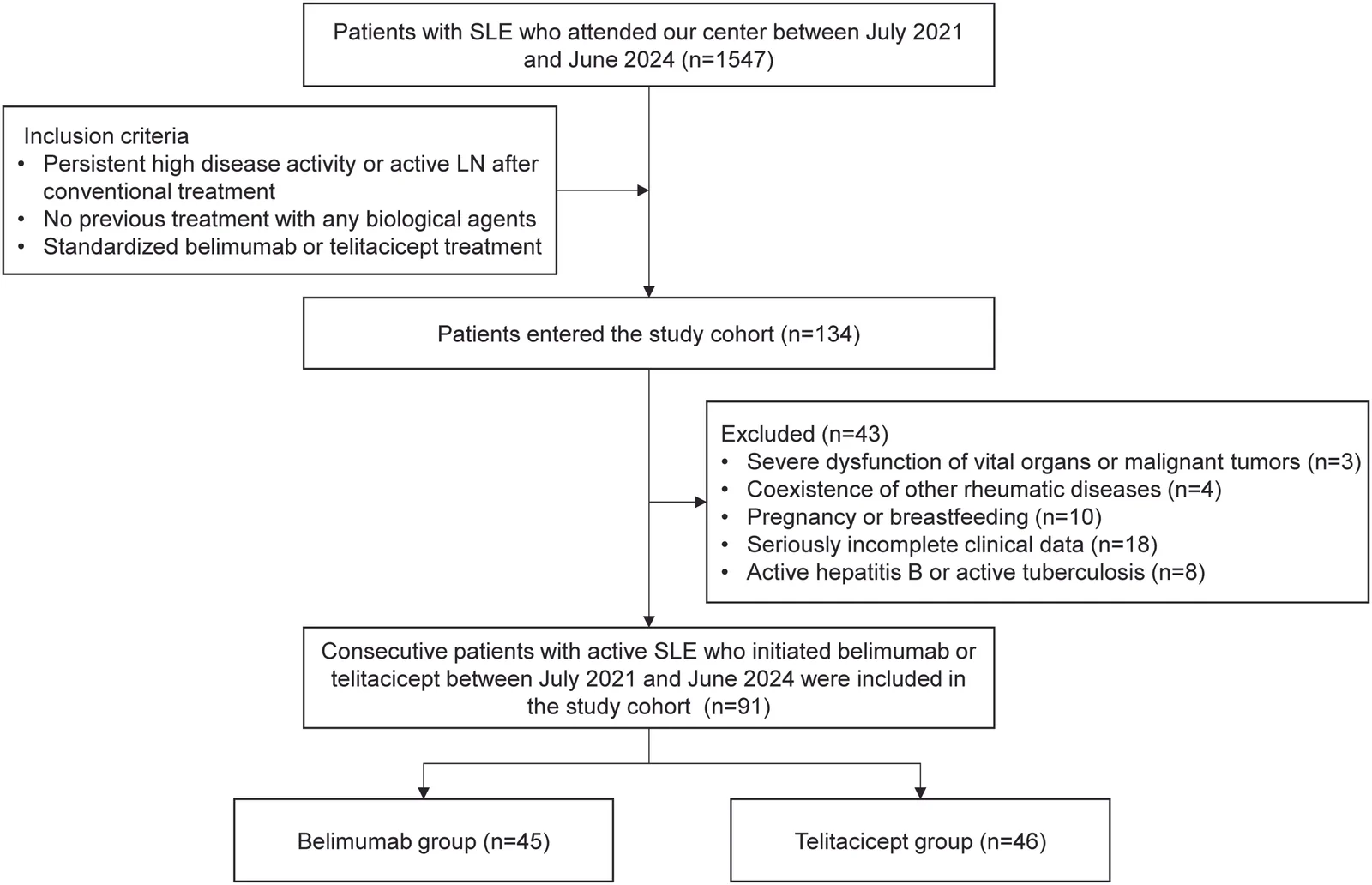
Study flow diagram.

**Table 1:** Baseline characteristics for patients with active SLE.

Characteristics	Before IPTW			After IPTW		
	Belimumab (n=45)	Telitacicept (n=46)	SMD	Belimumab (n=44.7)	Telitacicept (n=41.9)	SMD
Age, year	36.22 ± 11.49	34.48 ± 10.83	-0.156	34.54 ± 11.30	35.41 ± 10.08	0.004
Sex, n (%)			-0.297			-0.115
Male	4 (8.89)	1 (2.17)		2.4 (5.30)	1.3 (3.02)	
Female	41 (91.11)	45 (97.83)		42.3 (94.70)	40.6 (96.98)	
Disease duration, year	9 (4, 15)	10.5 (4.5, 16)	0.164	11 (5, 15)	12 (6, 15)	0.021
Serological indicators						
Anti-dsDNA, IU/mL	161.00 (61.10, 296.00)	71.30 (23.40, 542.60)	-0.095	161.00 (61.10, 407.00)	71.10 (23.40, 439.00)	-0.027
IgG, g/L	14.60 (10.60, 19.10)	13.70 (9.96, 17.20)	-0.135	13.10 (10.20, 19.10)	12.60 (9.36, 16.20)	-0.011
IgA, g/L	2.22 (1.60, 3.16)	2.38 (1.67, 3.68)	0.191	2.05 (1.47, 3.16)	2.24 (1.93, 3.40)	0.012
IgM, g/L	0.82 (0.55, 1.36)	1.12 (0.69, 1.60)	-0.060	0.83 (0.41, 1.35)	1.13 (0.71, 1.57)	0.012
C3, g/L	0.58 ± 0.21	0.65 ± 0.21	0.355	0.58 ± 0.20	0.65 ± 0.19	0.018
C4, g/L	0.10 (0.05, 0.13)	0.13 (0.07, 0.18)	0.465	0.09 (0.05, 0.13)	0.12 (0.05, 0.18)	0.047
Disease activity						
SLEDAI-2K	10 (8, 12)	9.5 (8, 12)	-0.022	10 (8, 12)	10 (8, 12)	0.002
PGA	2.4 (2, 2.8)	2.45 (2, 2.8)	0.057	2.5 (2, 2.8)	2.5 (2, 2.8)	0.001
Glucocorticoid use						
Prednisone at baseline, mg/day	20 (12.5, 32)	15 (10, 25)	-0.360	20 (12.5, 35)	15 (10, 25)	-0.041
Antimalarial drug, n (%)						
HCQ	38 (84.44)	37 (80.43)	0.106	38.1 (85.30)	30.7 (73.34)	0.299
Immunosuppressants, n (%)						
MMF	29 (64.44)	30 (65.22)	-0.016	29.3 (65.56)	24.1 (57.49)	0.166
LEF	3 (6.67)	1 (2.17)	0.220	4.6 (10.20)	0.6 (1.46)	0.380
FK506	3 (6.67)	1 (2.17)	0.220	4.3 (9.71)	0.8 (1.88)	0.340
CsA	0 (0)	1 (2.17)	-0.211	0 (0)	0.9 (2.18)	-0.211
CTX	0 (0)	1 (2.17)	-0.211	0 (0)	0.6 (1.35)	-0.166
MTX	4 (8.89)	2 (4.35)	0.183	3.4 (7.61)	1.3 (3.17)	0.197

Data are presented as mean ± SD, median (IQR) or n (%). IPTW, inverse probability of treatment weighting; C3, complement 3; C4, complement 4; SLEDAI-2K, systemic lupus erythematosus disease activity index 2000; PGA, physician’s global assessment; HCQ, hydroxychloroquine; MMF, mycophenolate mofetil; LEF, leflunomide; FK506, tacrolimus; CsA, cyclosporin A; CTX, cyclophosphamide; MTX, methotrexate.

To further adjust for potential confounding, we performed IPTW based on baseline covariates. A SMD of < 0.1 was considered indicative of adequate balance. After IPTW, most covariates achieved balance; however, sex and baseline immunosuppressant use remained imbalanced (SMD > 0.1, **Figure 2**).

**Figure 2:**
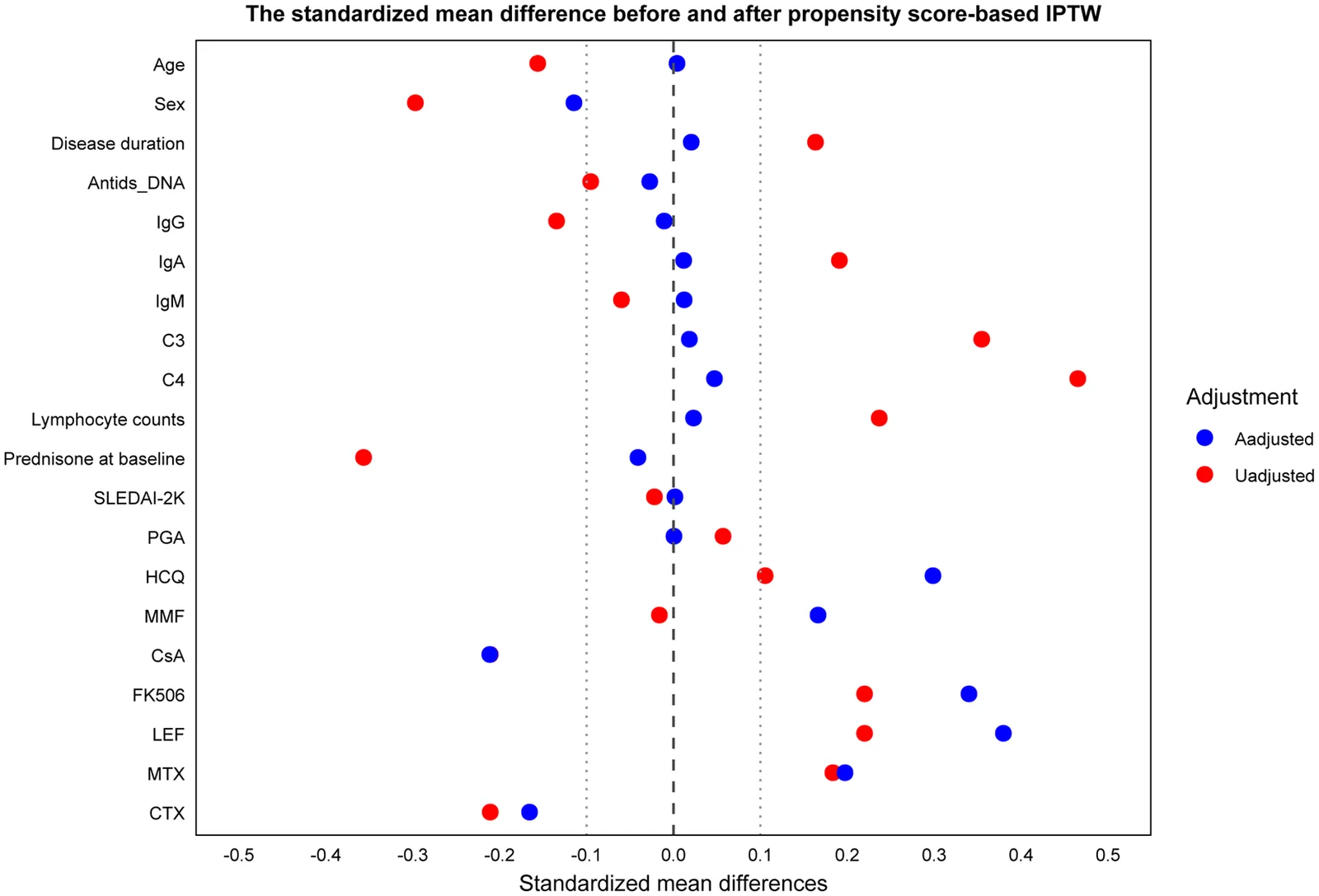
The standardized mean differences before and after propensity score-based inverse probability of treatment weighting. Standardized mean differences (SMD) of baseline characteristics between treatment groups are shown. Red dots indicate unadjusted SMD before IPTW; blue dots indicate adjusted SMD after IPTW. The dashed line at 0 indicates perfect balance; dotted lines at ±0.1 denote the threshold for acceptable balance.

### 3.2 Effectiveness outcomes after propensity score-based IPTW

As shown in **Figure 3A**, the proportion of patients achieving LLDAS after treatment with telitacicept was significantly higher than with belimumab (week 12: 8.9% vs. 1.13%, *P* = 0.027; week 24: 30.92% vs. 11.35%, *P* = 0.026). A sensitivity analysis was performed by including sex and baseline immunosuppressant use as covariates in the model comparing the 24-week LLDAS response between the two groups. After adjusting for these two variables, the difference remained statistically significant (*P* = 0.041), supporting the robustness of the primary finding (**Supplementary Table 2**).

**Figure 3:**
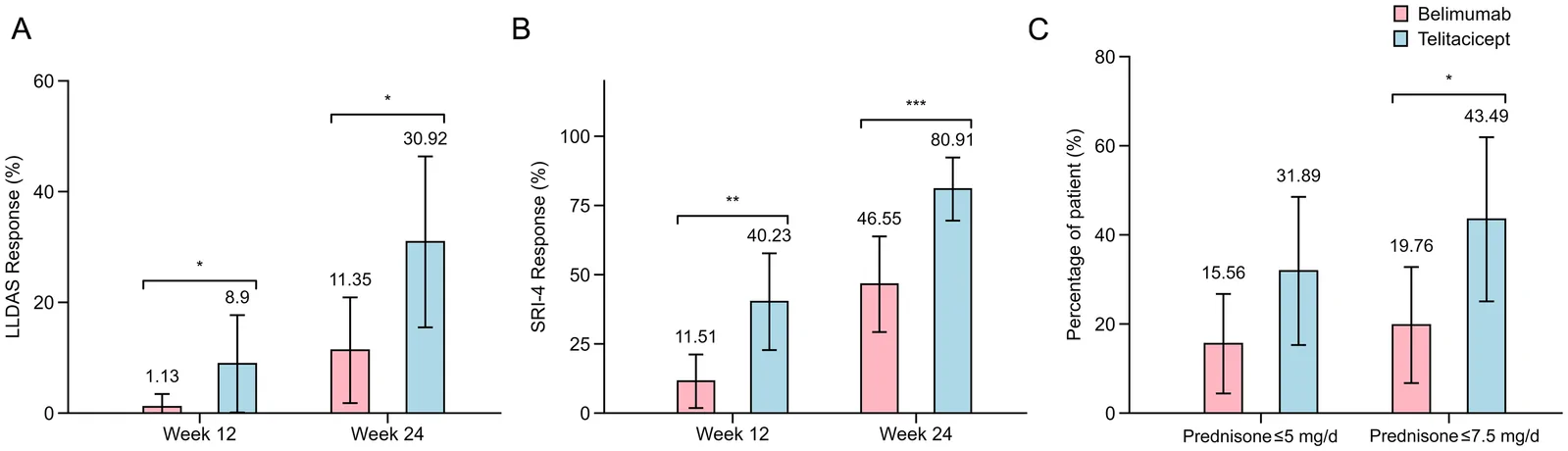
Clinical outcomes between belimumab and telitacicept treatment after IPTW. **(A)** LLDAS response at weeks 12 and 24. **(B)** SRI-4 response at weeks 12 and 24. **(C)** The proportion of patients achieving prednisone dose reduction to ≤5 mg/d and ≤7.5 mg/d at week 24. ^*^P < 0.05, ^**^P < 0.01, ^***^P < 0.001. Error bars represent 95% confidence intervals. Data are presented as weighted proportions; weighted denominators: telitacicept n = 41.9, belimumab n = 44.7.

For secondary outcomes, SRI-4 response rates were evaluated at weeks 12 and 24. Statistically significant differences between the two groups were observed at both time points, favoring the telitacicept group (all *P* < 0.01, **Figure 3B**). Specifically, at week 12, the SRI-4 response rate was 40.23% in the telitacicept group and 11.51% in the belimumab group; at week 24, the corresponding rates were 80.91% and 46.55%, respectively. The benefits of telitacicept extended to prednisone dose reduction as well. At week 24, after excluding patients who were already at or below the target dose at baseline, a significantly greater proportion of patients in the telitacicept group achieved glucocorticoid tapering to ≤7.5 mg/d compared with the belimumab group (43.49% vs. 19.76%, *P* = 0.03, **Figure 3C**). For tapering to ≤5 mg/d, the difference between the two groups was no longer statistically significant (31.89% vs. 15.56%, *P* = 0.09). Regarding the absolute change in glucocorticoid dose from baseline, no significant difference was observed between the two groups at week 24 [median change (IQR): -5.00 (-15.00, -2.50) vs. -10.00 (-20.00, -2.50); *P* = 0.34].

Regarding serological indicators, changes in C3 and C4 levels in the two patient groups at week 12 and 24 of treatment were shown in **Figures 4A, B**. Compared with baseline, both the belimumab group and the telitacicept group exhibited an increasing trend in C3 and C4 levels at each time point, but the differences between the two groups were not statistically significant (*P* > 0.05). Similarly, after 12 and 24 weeks of treatment, no statistically significant differences were observed in the levels of IgA, IgM, and IgG between the two groups (**Figures 4C–E**). The reduction in anti-dsDNA levels from baseline was comparable between the two groups (**Figure 4F**). Furthermore, the telitacicept group showed a significantly greater reduction in SLEDAI-2K score from baseline at weeks 12 and 24 (**Figure 3G**), reflecting better therapeutic effectiveness. The LSM [95% CI] of SLEDAI-2K score was significantly lower in the telitacicept group compared with the belimumab group (-3.2 [-3.6, -2.7] vs. -1.8 [-2.3, -1.4]; LSM difference, 1.3 [0.7, 2]; *P* < 0.001) at week 12. Similarly, at week 24, the LSM [95% CI] of SLEDAI-2K score remained significantly lower in the telitacicept group than in the belimumab group (-4.2 [-4.8, -3.6] vs. -3.1 [-3.7, -2.5]; LSM difference, 1.1 [0.2, 2]; *P* = 0.015).

**Figure 4:**
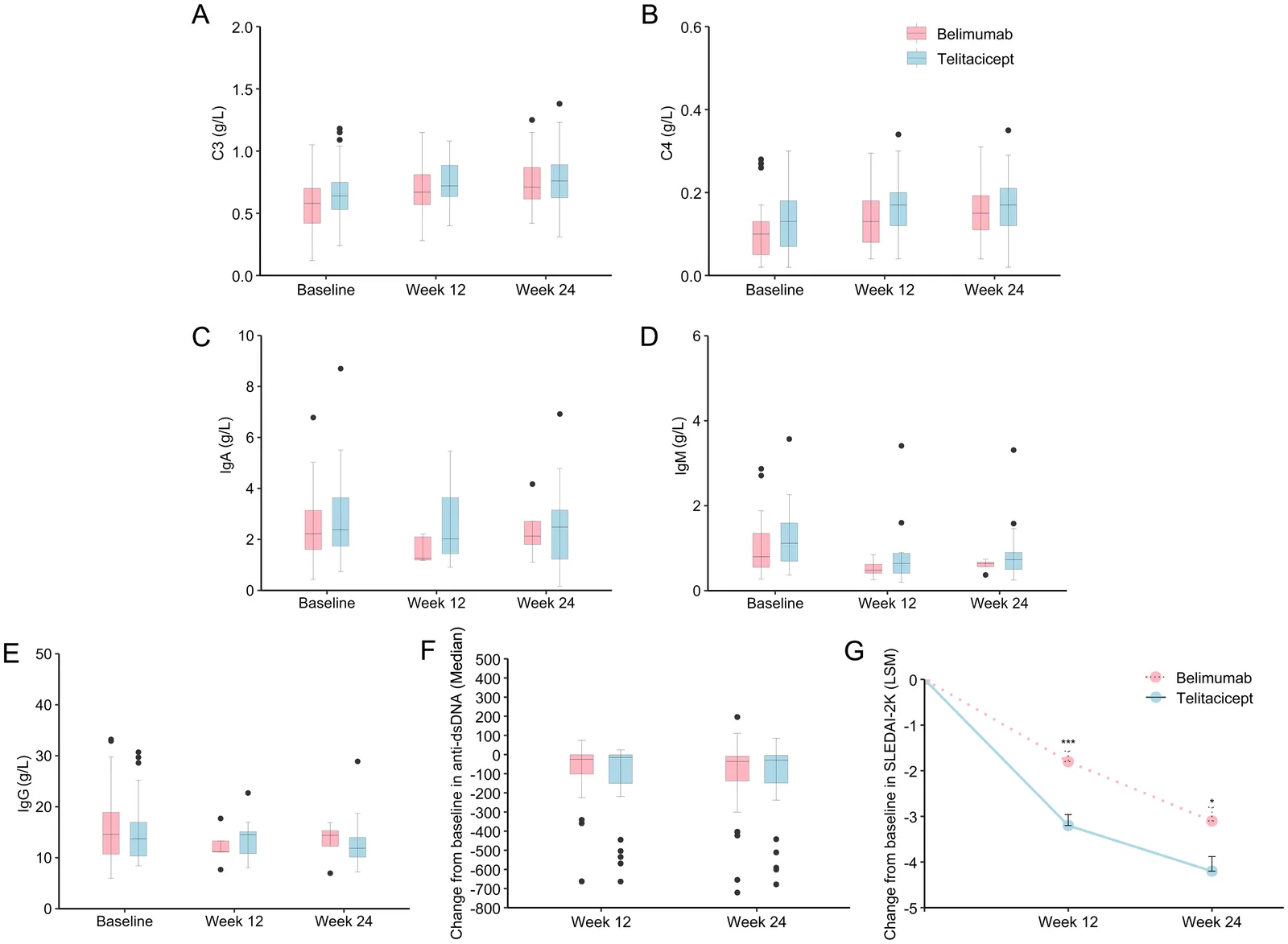
Changes in serological indicators and SLEDAI-2K score from baseline to 12 and 24 weeks between belimumab and telitacicept treatment after IPTW. Median changes in C3 **(A)** and C4 **(B)**. Mean changes in IgA **(C)**, IgM **(D)** and IgG **(E)**. Change from baseline in anti-dsDNA **(F)** and SLEDAI-2K **(G)** at weeks 12 and 24. ^*^P < 0.05, ^***^P < 0.001.

### 3.3 Subgroup analyses in patients with LN or hematological abnormalities

In this cohort, a total of 51 patients were diagnosed with LN, including 29 who received belimumab and 22 who received telitacicept. At week 24, the LLDAS response rate was significantly higher in the telitacicept group than in the belimumab group (23.94% vs. 2.14%, *P* = 0.005, **Figure 5A**). Regarding SRI-4 response rates, the telitacicept group also demonstrated superior outcomes (**Figure 5B**), with statistically significant between-group differences observed at both week 12 (52.77% vs. 10.16%, *P* < 0.001) and week 24 (85.49% vs. 51.18%, *P* < 0.01). However, no statistically significant differences were observed between the two groups in the change of CRR, PRR, 24hUP, and total UP (all *P* > 0.05, **Figure 5C**). Among patients with hematological abnormalities, defined as hemoglobin (belimumab, n=15; telitacicept, n=8), platelet count (belimumab, n=2; telitacicept, n=3), or white blood cell count (belimumab, n=11; telitacicept, n=6) below the lower limit of normal. After 24 weeks of treatment, the two groups showed comparable recovery in hemoglobin, platelet count, and white blood cell count (all *P* > 0.05).

**Figure 5:**
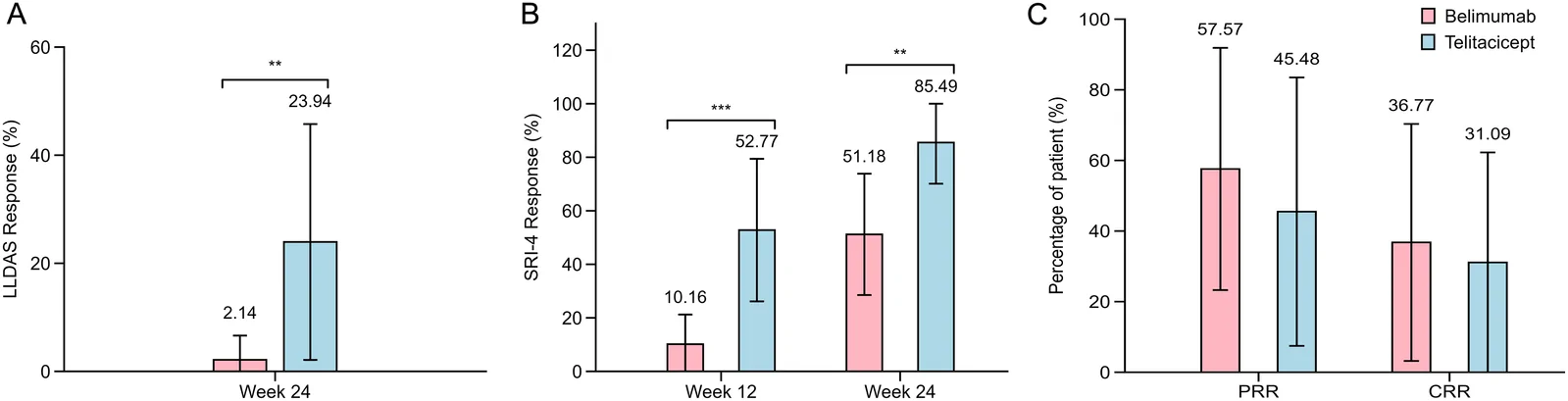
Treatment outcomes in lupus nephritis subgroup after IPTW. **(A)** LLDAS response at week 24 in lupus nephritis subgroup. **(B)** SRI-4 response at weeks 12 and 24 lupus nephritis subgroup. **(C)** Comparison of the proportion of lupus nephritis patients achieving PRR and CRR. ^**^P < 0.01, ^***^P < 0.001. Error bars represent 95% confidence intervals. Data are presented as weighted proportions; weighted denominators: telitacicept n = 19.5, belimumab n = 13.7.

### 3.4 Exploratory analysis of factors associated with SRI-4 response

Univariate logistic regression analysis of variables identified treatment with telitacicept (OR = 4.876, *P* = 0.001), disease duration (OR = 0.916, *P* = 0.048) and baseline SLEDAI-2K score (OR = 1.312, *P* = 0.012) as significant associated with SRI-4 response at week 24 (**Table 2**). Both factors remained significant in the multivariate logistic regression model (treatment with telitacicept: OR = 4.975, *P* = 0.006; disease duration: OR = 0.914, *P* = 0.031; SLEDAI-2K: OR = 1.316, *P* = 0.016).

**Table 2:** Factors associated with SRI‐4 response based on univariate and multivariate logistic regression analysis.

Parameters	Univariate, OR (95%CI)	*P* value	Multivariate, OR (95%CI)	*P* value
Treatment				
Belimumab	1	/	1	/
Telitacicept	4.876 (1.847, 12.825)	**0.001**	4.975 (1.568, 15.778)	**0.006**
Age	1.006 (0.965, 1.049)	0.769	/	/
Sex	0.826 (0.087, 7.841)	0.868	/	/
Disease duration	0.916 (0.839, 0.999)	**0.048**	0.914 (0.843, 0.992)	**0.031**
Height	1.084 (0.990, 1.187)	0.081	/	/
Weight	1.034 (0.991, 1.079)	0.123	/	/
SLEDAI-2K	1.312 (1.062, 1.620)	**0.012**	1.316 (1.054, 1.645)	**0.016**
PGA	1.529 (0.551, 4.240)	0.415	/	/
TC	1.069 (0.722, 1.583)	0.738	/	/
TG	0.825 (0.434, 1.569)	0.558	/	/
B cell	1.015 (0.958, 1.076)	0.615	/	/
WBC	0.823 (0.669, 1.011)	0.064	/	/
HB	0.991 (0.962, 1.021)	0.548	/	/
PLT	1.006 (0.999, 1.013)	0.112	/	/
Neutrophil	0.823 (0.655, 1.057)	0.132	/	/
Lymphocyte	0.720 (0.444, 1.169)	0.184	/	/
ESR	1.026 (0.998, 1.055)	0.072	/	/
24 h urinary protein	1.012 (0.720, 1.421)	0.947	/	/
GFR	1.007 (0.993, 1.022)	0.336	/	/
Urinary total protein	0.992 (0.981, 1.003)	0.173	/	/
AST	0.993 (0.955, 1.033)	0.742	/	/
ALT	0.987 (0.965, 1.009)	0.253	/	/
Cr	0.988 (0.973, 1.003)	0.121	/	/
C3	1.224 (0.112, 13.410)	0.869	/	/
IgG	1.032 (0.943, 1.130)	0.488	/	/
IgA	1.007 (0.677, 1.499)	0.972	/	/
IgM	1.169 (0.631, 2.166)	0.620	/	/
Anti-dsDNA	1.000 (0.999, 1.001)	0.946	/	/
LN	0.534 (0.196, 1.007)	0.677		
Prednisone	1.033 (0.992, 1.076)	0.117	/	/
Immunosuppressants	0.525 (0.160, 1.720)	0.287	/	/

OR, odds ratio; CI, confidence interval; SLEDAI-2K, systemic lupus erythematosus disease activity index 2000; PGA, physician’s global assessment; TC, total cholesterol; TG, triglyceride; WBC, white blood cell; HB, hemoglobin; PLT, platelet; ESR, erythrocyte sedimentation rate; GFR, glomerular filtration rate; AST, aspartate aminotransferase; ALT, alanine aminotransferase; Cr, creatinine; C3, complement 3; IgG, immunoglobulin G; IgA, immunoglobulin A; IgM, immunoglobulin M; Anti-dsDNA, anti-double-stranded DNA.

*P* values in bold indicate statistical significance (*P*<0.05).

### 3.5 Safety outcomes

During the 24-week observation period, the overall incidence of AEs was low in both groups (**Table 3**). Specifically, AEs occurred in 6.7% (3/45) of patients in the belimumab group and 8.7% (4/46) in the telitacicept group, with no severe AEs reported. Infection was the most common AE, occurring in two patients in each group. Upper respiratory infection was the most frequent (n = 2), followed by cytomegalovirus infection (n = 1) and urinary tract infection (n = 1). All infectious events resolved after anti-infective treatment and symptomatic supportive care. Non-infectious events included injection site reaction (n = 1) and liver function abnormality (n = 2); all were transient and resolved with symptomatic management. No treatment discontinuation or dose adjustment due to AEs was observed.

**Table 3:** Adverse events for patients with active SLE after 24 weeks treatment with belimumab or telitacicept.

AEs, n (%)	Belimumab (n=45)	Telitacicept (n=46)
Any AEs	3 (6.7)	4 (8.7)
Serious AEs	0 (0)	0 (0)
Injection site reaction	0 (0)	1 (2.2)
Upper respiratory infection	0 (0)	2 (4.3)
Liver function abnormality	1 (2.2)	1 (2.2)
Cytomegalovirus infection	1 (2.2)	0 (0)
Urinary tract infection	1 (2.2)	0 (0)

Data are presented as n (%). AEs, adverse events.

## 4 Discussion

LLDAS is a core treatment target in SLE, and its achievement is closely associated with slowed progression of organ damage, reduced risk of severe relapse, and decreased mortality (21). In this retrospective cohort study comparing telitacicept and belimumab in patients with active SLE, we found that telitacicept was associated with a higher rate of LLDAS compared with belimumab (30.92% vs. 11.35%) at week 24. This difference was consistent with a recent retrospective observational study, which reported numerically higher LLDAS achievement rates with telitacicept than with belimumab at week 24 (22.9% vs. 16.5%) (22). Additionally, our data also revealed that telitacicept provided a numerically higher SRI-4 response over belimumab at week 24 (80.91% vs. 46.55%), which is consistent with findings from a previous Chinese SLE cohort study (22). Collectively, these consistent results across independent real-world studies support the potential advantage of dual BLyS/APRIL blockade over BLyS monotherapy in achieving both LLDAS and SRI-4 responses in active SLE.

Minimizing glucocorticoid exposure is a key therapeutic goal in SLE management, as sustained use is associated with irreversible organ damage, including avascular necrosis, osteoporosis, and accelerated atherosclerosis (23). Both telitacicept and belimumab have been shown individually to reduce glucocorticoid burden in patients with active SLE (9, 17). To date, two head-to-head studies have compared telitacicept and belimumab in active SLE, requiring SLEDAI-2K scores of ≥4 and ≥6 for patient inclusion, respectively (11, 22). Our study complemented these findings by focusing on a patient population with higher disease burden, as reflected by the entry criteria of SLEDAI-2K ≥8 or active LN. This broader and more severe spectrum of SLE—including patients with high systemic disease activity or active renal involvement—has not been adequately represented in previous comparative studies. A significant between-group difference in favor of telitacicept was limited to the ≤7.5 mg/d tapering endpoint; the difference did not reach statistical significance for the ≤5 mg/d threshold, and the absolute reduction in glucocorticoid dose was similar between the two groups. This pattern implies that the advantage of telitacicept over belimumab in steroid tapering may be concentrated at a specific clinically relevant threshold, rather than representing a broadly greater tapering effect across all measures.

Although no significant between-group differences were observed in complement or immunoglobulin levels, the divergent trends in immunoglobulin trajectories—an initial decline followed by a rebound in the belimumab group versus a sustained decline in the telitacicept group—may reflect differences in their mechanisms of action. Belimumab neutralizes only soluble BLyS, whereas telitacicept also inhibits APRIL, which is important for long-lived plasma cell survival (16). Whether this observed trend translates into differential long-term clinical outcomes warrants further investigation. Both groups showed a low number of documented AEs (6.7% for belimumab vs. 8.7% for telitacicept), and no serious AEs or safety-related discontinuations were recorded. However, given the small number of events, these safety findings should be interpreted descriptively, and no formal between-group comparisons or definitive conclusions can be made.

Renal involvement, the most common visceral manifestation of SLE, is a key predictor of poor prognosis and represents the leading cause of death associated with the disease (24). In this SLE cohort, renal abnormalities were highly prevalent, affecting a considerable proportion of patients (56%). Our data showed that telitacicept achieved significantly higher LLDAS and SRI-4 response rates compared with belimumab in the LN subgroup, consistent with the findings in the overall cohort. These findings pointed to a potential advantage of telitacicept in both lowering overall disease activity and attaining the low disease activity state among patients with LN. However, no significant between-group differences were observed in objective renal measures, including CRR, PRR, 24hUP, or total UP. One possible explanation is that the superior performance of telitacicept on LLDAS and SRI-4—composite endpoints that capture disease activity across multiple organ systems—may be attributable to its broader effects on non-renal manifestations, such as mucocutaneous and musculoskeletal involvement, rather than to differential kidney-specific efficacy (25). Additionally, the small sample size and relatively short follow-up duration may have limited the statistical power to detect differences in objective renal outcomes, as complete renal response typically requires 6–12 months or longer to manifest (26). Future prospective, randomized studies with larger cohorts and extended observation periods are warranted to further explore this question.

In the exploratory multivariable analysis, treatment with telitacicept, baseline SLEDAI-2K score and disease duration were independently associated with SRI-4 response at week 24. Similar observations were also reported in the clinical trials of belimumab, where the response rate of SRI-4 was higher in patients with a baseline SELENA-SLEDAI score of ≥10 compared to those with a score of ≤9 (27). This finding implies that, in SLE patients treated with B-cell-targeted biologics, higher baseline disease activity may be associated with a greater margin of therapeutic benefit. Notably, longer disease duration was associated with a significantly lower odds of achieving SRI-4 response (OR = 0.914, *P* = 0.031), highlighting that delayed treatment initiation may compromise therapeutic efficacy. The evolving treatment paradigm for SLE, as reflected in the EULAR 2025 recommendations for the management of SLE with kidney involvement, supports earlier integration of biologic agents into the therapeutic algorithm, without requiring prior failure of conventional immunosuppressive drugs (28). Given the observational design and limited sample size, these exploratory findings regarding the factors associated with SRI-4 response should be interpreted with caution and warrant validation in larger prospective cohorts. In addition, we acknowledge that similar analyses for LLDAS attainment did not yield significant associated factors; this may be attributable to the smaller number of LLDAS responders, which limited the statistical power for detecting such associations.

Several limitations of this study should be acknowledged. First, the retrospective design was inherently subject to selection bias and information bias. Although we applied IPTW to minimize confounding, residual confounding may still exist, as evidenced by the persistent imbalance in sex and baseline immunosuppressant use after weighting. Second, the relatively small sample size limited statistical power, particularly for subgroup analyses in patients with LN or hematologic involvement. Third, the 24-week follow-up period limited the evaluation of long-term clinical outcomes and safety. Fourth, our analysis was restricted to patients who completed at least 24 weeks of continuous treatment with adequate follow-up data; patients who lacked post-baseline assessments due to financial constraints were excluded. Consequently, our findings are strictly applicable to patients who tolerate and adhere to at least 24 weeks of biologic therapy and should not be overgeneralized to all SLE patients initiating biologics in real-world practice. Finally, as a single-center study, our findings may not be generalizable to other populations. Therefore, larger, multicenter, prospective studies with extended follow-up are warranted to confirm our observations.

## 5 Conclusion

After IPTW adjustment, telitacicept showed a statistically significant association with higher LLDAS and SRI-4 response rates in this single-center retrospective cohort of patients with active SLE who completed 24 weeks of treatment. The small number of AEs limits any comparative safety assessment. These findings should be considered preliminary and warrant confirmation in prospective randomized studies.

## Data Availability

The original contributions presented in the study are included in the article/**Supplementary Material**. Further inquiries can be directed to the corresponding authors.
