# Supplementary material for: Effectiveness and safety of belimumab and telitacicept in active systemic lupus erythematosus: a retrospective cohort study
Source: Front Immunol. 2026 Sep 21;17:1927886. doi: 10.3389/fimmu.2026.1927886 (PMC13635200; doi:10.3389/fimmu.2026.1927886)
Supplement: Supplementary file 1 [file SupplementaryFile1.docx]

***Supplementary Materials***

1 **Supplementary Data**

**Propensity score‑based IPTW**

To account for potential confounding by baseline characteristics, we performed inverse probability of treatment weighting (IPTW) using propensity scores. Propensity scores were estimated from a multivariable logistic regression model with treatment assignment (telitacicept vs. belimumab) as the outcome and the following baseline covariates as predictors: age, sex, SLEDAI-2K, PGA and BILAG. Treatment assignment coded as 1 for telitacicept and 0 for belimumab. No missing data were present for any of these five covariates. Stabilized inverse probability of treatment weights (SIPTW) were constructed to mitigate the impact of extreme weights. For patients receiving telitacicept, weights were calculated as SW = P (ARM = 1) / PS; for those receiving belimumab, weights were calculated as SW = P (ARM = 0) / (1 − PS). The raw propensity scores ranged from 0.1365 to 0.9368 in the telitacicept group and from 0.0046 to 0.8106 in the belimumab group. The corresponding stabilized weight ranges were 0.5396-3.7034 for telitacicept and 0.4968-2.6107 for belimumab. To ensure adequate overlap in PS distributions between the two groups, we restricted the analysis to the region of common support, defined as the overlapping PS interval of 0.1365-0.8106; observations outside this region were excluded from all weighted analyses. The nominal weighted sample sizes (sum of stabilized weights) were 41.9 for telitacicept and 44.7 for belimumab, with effective sample sizes of 35.8 and 34.5, respectively.

2 **Supplementary Tables**

**Supplementary Table 1** Missing data proportions for each variable at each time point

| variable | Baseline | Week 12 | Week 24 |
| --- | --- | --- | --- |
| Age | 0 (0.0%) | 0 (0.0%) | 0 (0.0%) |
| Sex | 0 (0.0%) | 0 (0.0%) | 0 (0.0%) |
| Height | 11 (12.1%) | 11 (12.1%) | 11 (12.1%) |
| Weight | 11 (12.1%) | 11 (12.1%) | 11 (12.1%) |
| Disease duration | 14 (15.4%) | 14 (15.4%) | 14 (15.4%) |
| B cell | 34 (37.4%) | 34 (37.4%) | 34 (37.4%) |
| TC | 34 (37.4%) | 34 (37.4%) | 34 (37.4%) |
| WBC | 0 (0.0%) | 2 (2.2%) | 0 (0.0%) |
| HB | 1 (1.1%) | 2 (2.2%) | 0 (0.0%) |
| PLT | 1 (1.1%) | 2 (2.2%) | 0 (0.0%) |
| Neutrophil | 1 (1.1%) | 3 (3.3%) | 2 (2.2%) |
| ESR | 3 (3.3%) | 3 (3.3%) | 0 (0.0%) |
| 24 h urinary protein | 12 (13.2%) | 21 (23.1%) | 31 (34.1%) |
| AST | 5 (5.5%) | 8 (8.8%) | 5 (5.5%) |
| ALT | 6 (6.6%) | 8 (8.8%) | 5 (5.5%) |
| Cr | 19 (20.9%) | 38 (41.8%) | 29 (31.9%) |
| C3 | 1 (1.1%) | 7 (7.7%) | 4 (4.4%) |
| C4 | 1 (1.1%) | 7 (7.7%) | 4 (4.4%) |
| Anti-dsDNA | 3 (3.3%) | 12 (13.2%) | 8 (8.8%) |
| IgG | 35 (38.5%) | 73 (80.2%) | 67 (73.6%) |
| IgA | 35 (38.5%) | 73 (80.2%) | 67 (73.6%) |
| IgM | 35 (38.5%) | 73 (80.2%) | 67 (73.6%) |
| SLEDAI-2K | 0 (0.0%) | 0 (0.0%) | 0 (0.0%) |
| BILAG | 0 (0.0%) | 0 (0.0%) | 0 (0.0%) |
| PGA | 0 (0.0%) | 0 (0.0%) | 0 (0.0%) |
| Prednisone | 0 (0.0%) | 0 (0.0%) | 0 (0.0%) |

Abbreviation: SLEDAI-2K, systemic lupus erythematosus disease activity index 2000; PGA, physician’s global assessment; TC, total cholesterol; TG, triglyceride; WBC, white blood cell; HB, hemoglobin; PLT, platelet; ESR, erythrocyte sedimentation rate; GFR, glomerular filtration rate; AST, aspartate aminotransferase; ALT, alanine aminotransferase; Cr, creatinine; C3, complement 3; IgG, immunoglobulin G; IgA, immunoglobulin A; IgM, immunoglobulin M; Anti-dsDNA, anti-double-stranded DNA.

**Supplementary Table 2 Analysis of LLDAS logistic regression.**

| **Visit** | **Variable** | **OR (95%CI)** | ***P* value** |
| --- | --- | --- | --- |
| Week 12 | Treatment group | 9.02 (0.43, 188.04) | 0.156 |
|  | Sex | 0.16 (0.01, 5.25) |  |
|  | Immunosuppressants | 0.57 (0.07, 4.58) |  |
| Week 24 | Treatment group | 3.32 (1.05, 10.44) | 0.040 |
|  | Sex | 1.27 (0.06, 28.68) |  |
|  | Immunosuppressants | 0.73 (0.23, 2.28) |  |

Note: Sensitivity analysis adjusting for residual imbalance in sex and immunosuppressant use after IPTW. Abbreviation: OR, odds ratio; CI, confidence interval.
